# Carriage of extended-spectrum beta-lactamase-producing Enterobacteriaceae in HIV-infected children in Zimbabwe

**DOI:** 10.1099/jmm.0.000474

**Published:** 2017-05-18

**Authors:** S. M. S Wilmore, K Kranzer, A Williams, B Makamure, A. F Nhidza, J Mayini, T Bandason, J Metcalfe, M. P Nicol, I Balakrishnan, M. J Ellington, N Woodford, S Hopkins, T. D McHugh, R. A Ferrand

**Affiliations:** ^1^​ UCL Centre for Clinical Microbiology, University College London, London, UK; ^2^​ Royal Free Hospital NHS Trust, London, UK; ^3^​ London School of Hygiene and Tropical Medicine, London, UK; ^4^​ National German Mycobacterium Reference, Borstel, Germany; ^5^​ Biomedical Research and Training Institute, Harare, Zimbabwe; ^6^​ University of California, San Francisco, USA; ^7^​ University of Cape Town, National Health Laboratory Service, Cape Town, South Africa; ^8^​ Public Health England, London, UK

**Keywords:** antimicrobial resistance, Africa, extended-spectrum, beta-lactamase Enterobacteriaceae, ESBL, HIV

## Abstract

**Background:**

Antimicrobial resistance is an emerging global health issue. Data on the epidemiology of multidrug-resistant organisms are scarce for Africa, especially in HIV-infected individuals who often have frequent contact with healthcare. We investigated the prevalence of extended-spectrum beta-lactamase-producing Enterobacteriaceae (ESBL-E) carriage in stool among HIV-infected children attending an HIV outpatient department in Harare, Zimbabwe.

**Methods:**

We recruited children who were stable on antiretroviral therapy (ART) attending a HIV clinic from August 2014 to June 2015. Information was collected on antibiotic use and hospitalization. Stool was tested for ESBL-E through combination disc diffusion. API20E identification and antimicrobial susceptibility was performed on the positive samples followed by whole genome sequencing.

**Results:**

Stool was collected from 175/202 (86.6 %) children. Median age was 11 [inter-quartile range (IQR) 9–12] years. Median time on ART was 4.6 years (IQR 2.4–6.4). ESBL-Es were found in 24/175 samples (13.7 %); 50 % of all ESBL-Es were resistant to amoxicillin-clavulanate, 100 % to co-trimoxazole, 45.8 % to chloramphenicol, 91.6 % to ceftriaxone, 20.8 % to gentamicin and 62.5 % to ciprofloxacin. ESBL-Es variously encoded CTX-M, OXA, TEM and SHV enzymes. The odds of ESBL-E carriage were 8.5 times (95 % CI 2.2–32.3) higher in those on ART for less than one year (versus longer) and 8.5 times (95 % CI 1.1–32.3) higher in those recently hospitalized for a chest infection.

**Conclusion:**

We found a 13.7 % prevalence of ESBL-E carriage in a population where ESBL-E carriage has not been described previously. Antimicrobial resistance (AMR) in Africa merits further study, particularly given the high HIV prevalence and limited diagnostic and therapeutic options available.

## Introduction

The widespread use of antibiotics worldwide has resulted in the emergence of a global problem of antimicrobial resistance (AMR). Over the past decade, there has been an increasing prevalence of carriage of and infection with multidrug-resistant organisms (MDROs) such as extended-spectrum beta-lactamase-producing Enterobacteriaceae (ESBL-E) and carbapenemase-producing Enterobacteriaceae (CPE) [1]. ESBL and carbapenemase enzymes are most commonly located on plasmids and transposons, which can facilitate transmission of resistance within and between species, and carriage therefore presents an infection control risk particularly within hospitals. Mobile genetic elements (MGEs) are a type of resistance encoded by a plasmid which can transfer antibiotic resistance to different bacteria and are increasingly recognized as an additional infection control risk within healthcare settings. In the community, a French study reported a 10-fold increase in ESBL-E stool carriage from 0.6 % in 2006 to 6 % in 2011 among healthy volunteers attending a free five-yearly medical check-up programme [2]. The prevalence of ESBL-E rectal carriage in a more recent study of French children on routine check-ups at home by community paediatricians doubled between 2010 and 2015, from 4.8 to 10.2 % [3].

The emerging global threat of antimicrobial resistance (AMR) is particularly concerning for developing countries, as infections with ESBL-E and CPE are difficult to treat due to limited antibiotic treatment options that are not readily available in most resource-limited settings.

While the epidemiology, phenotypic and genotypic characteristics of MDROs are well described in Europe, North America and Asia [1], such data are scarce for Sub-Saharan Africa [4, 5]. African studies reporting on the prevalence of ESBL-E and CPE are mainly in the context of infection rather than carriage [5–8]. A study from the Central African Republic estimated the prevalence of ESBL-E in faeces of healthy children to be 59 % (79/134). CTX-M-15 was found in 81/83 (97.6 %) strains isolated and 39/51 (76 %) of the isolated *Escherichia coli* isolated pertained to the pandemic ST131 group [9].

Sub-Saharan Africa is the epicentre of the HIV epidemic, hosting an estimated 70 % (approximately 25 million) of the world’s HIV-infected population in 2014 [10]. Individuals living with HIV infection are at particular risk of acquiring MDROs due to the use of antibiotic prophylaxis and a high incidence of bacterial infections necessitating frequent antibiotic use. However, there is a dearth of studies investigating AMR carriage in the context of HIV infection. A study of 203 HIV-positive children recruited from outpatient clinics in Cape Town documented, in 2008, a 14.8 % nasopharyngeal carriage rate of Enterobacteriaceae, 50 % of which were ESBL-Es [11]. It is not yet known how carriage of these resistant bacteria from such a young age might affect the epidemiology of MDROs in this population as it grows older. Given the economic, environmental and healthcare-setting diversities between countries in Sub-Saharan Africa, it is important to obtain local epidemiological data on MDROs to develop strategies to address this growing problem.

The aim of our study was to determine the prevalence of ESBL-E and CPE carriage in stool among HIV-infected children accessing outpatient HIV care in Harare, Zimbabwe.

## Methods

### Study population

This study was conducted at the paediatric HIV care clinic at Harare Central Hospital, the largest public sector hospital in Harare, Zimbabwe. The clinic provides HIV care to over 3000 children. This study was nested in a larger study investigating the epidemiology of HIV-associated chronic lung disease (INHALE study). Perinatally HIV-infected children aged between 6 to 16 years who had been taking antiretroviral therapy (ART) for at least 6 months and were not acutely unwell were recruited consecutively over a 10-month period, with guardian consent and participant assent. Ethical approval for the study and shipment of specimens was granted by the Medical Research Council of Zimbabwe and the Research Council of Zimbabwe.

### Study procedures

A questionnaire recording socio-demographic details and clinical history, including duration of ART use and previous hospitalization, was administered on site to all participants/guardians. CD4 count was measured using the Alere PIMA analyser (Alere Technologies, Jena, Germany) and HIV viral load was measured using the COBAS Ampliprep/Taqman 48 version 2.0 (Roche Molecular Systems, Branchburg, USA). Participants were asked to provide a fresh stool sample on site or at home on the morning of the second study visit (INHALE study), using a stool collection kit and transported on ice if the participant had a freezer.

### Laboratory procedures

Freshly collected stool samples were inoculated onto cysteine lactose electrolyte-deficient (CLED) medium with the addition of a cefpodoxime disc (as indicator cephalosporin) for ESBL-E screening, and an ertapenem disc for CPE screening. Each colonial morphotype of oxidase-negative Gram-negative isolates that grew within a diameter zone of inhibition of ≤20 mm around the cefpodoxime disc or ≤28 mm around the ertapenem disc was subcultured for ESBL-E and CPE screening, respectively, and based on British Society for Antimicrobial Chemotherapy (BSAC) breakpoints [12]. ESBL-E detection was performed using combination disc diffusion testing following guidelines issued by Public Health England (PHE) [13]. The following discs were used and comparisons were made: cefpodoxime 30 µg±clavulanic acid and cefepime 30 µg±clavulanic acid (looking for a change in diameter zone ≥5 mm for ESBL confirmation). Organisms showing resistance to cefpodoxime without clavulanic acid synergy and susceptibility to cefepime were assumed to carry AmpC enzymes (or other inhibitor-stable beta-lactamases) and, given the identity of the organisms (*E. coli*), any AmpC genes would be likely to be plasmid-associated.

Preliminary identification of these resistant organisms was performed using API20E (BioMérieux, Lyon, France) and was subsequently confirmed using MALDI-TOF (MALDI Biotyper, Bruker, Bremen, Germany). Antimicrobial susceptibility testing (AST) was performed by disc diffusion testing following EUCAST methodology [14]. Isolates were tested against a range of antibiotics including those most commonly found in the Zimbabwean public health sector (amoxicillin-clavulanate, co-trimoxazole, chloramphenicol, ceftriaxone, trimethoprim, gentamicin and ciprofloxacin), those restricted to the private sector (piperacillin-tazobactam and meropenem) and those not known to be used in Zimbabwe (amikacin and temocillin). Disc diffusion as a method for ESBL-E screening and detection is recommended by the following agencies: Centres for Disease Control and Prevention (CDC), Clinical and Laboratory Standards Institute (CLSI) [15], as well as PHE and the Advisory Committee on Antimicrobial Resistance and Healthcare Associated Infection (ARHAI) [13].

### DNA extraction and sequencing

DNA was extracted from RNase-treated lysates using a Qiasymphony DSP DNA midi kit (Qiagen, Hilden, Germany). DNA libraries were prepared using the NexteraXT sample preparation method and sequenced with a standard 2×101 base protocol on a HiSeq 2500 Instrument (Illumina, San Diego, CA, USA). Sequence data from the sequencing runs were deplexed and trimmed, and the presence of antimicrobial resistance genes was determined with ‘Genefinder’ as described previously [16]. Genome assembly was performed using VelvetOptimiser with *k*-mer values from 47 to 71. blast searching against multi-locus sequence type (MLST) databases was used to identify the nearest match for each allele, followed by assignation of allelic profiles.

### Data analysis

Data were entered using Cardiff TELEFORM Intelligent Character Optical Mark Recognition Software (version 10.7) and analysed using STATA Version 13.1 (StataCorp, Texas, USA). Descriptive statistics were performed on the demographic and baseline characteristics of the participants. For continuous variables, non-parametric medians and inter-quartile ranges (IQRs) were calculated and for categorical variables, frequencies and percentages were determined. A χ^2^ test or Fisher’s exact (or non-parametric Mann–Whitney) test and univariate logistic regression were used to assess the association between the variables of interest and the occurrence of ESBL-E. A two-tailed *P*-value of <0.05 was considered to represent a statistically significant association. All the variables were considered regardless of their initial *P*-value into a multivariate logistic regression to assess the adjusted net effect of the independent variables.

## Results

A total of 202 eligible HIV-infected participants were recruited between August 2014 and June 2015, of whom 175/202 (86.6 %) were able to provide a stool sample and were included in this study. The median age was 11 (IQR 9–12) years and 48 % were female. The median time on ART was 4.6 years (IQR 2.4–6.4) (Table 1) and 96 % of participants were taking long-term prophylactic co-trimoxazole. The median CD4 count was 710 (IQR 468–952) cells µl^−1^, and 67.0 % of the participants had a viral load of <50 copies ml^−1^. Only 2 % of the participants had been admitted to the hospital for a chest infection/pneumonia in the previous 12 months.

**Table 1.** Demographic and clinical characteristics

	All (*n*=175)	ESBL-E negative (*n*=151)	ESBL-E positive (*n*=24)	*P*-value
**Demographics**				
Gender				
Male	91 (52.0 %)	80 (53.0 %)	11 (45.8 %)	
Female	84 (48.0 %)	71 (47.0 %)	13 (54.2 %)	0.515
Age (years)				
6–11	113 (64.6 %)	99 (65.6 %)	14 (58.3 %)	
12–16	62 (35.4 %)	52 (34.4 %)	10 (41.7 %)	0.492
Median age (IQR) years	11 (9–12)	11 (8–12)	11 (9–13)	0.291
Current CD4 count (cells µl^−1^)				
<350	19 (10.9 %)	15 (9.9 %)	4 (16.7 %)	0.325
≥350	156 (89.1 %)	136 (90.1 %)	20 (83.3 %)	
Median CD4 count (IQR)	710 (468–952)	710 (472–952)	699 (409–944)	0.548
Current viral load (copies ml^−1^)				
≤50	117 (66.8 %)	101 (66.9 %)	16 (66.7 %)	
>50	57 (32.6 %)	49 (32.5 %)	8 (33.3 %)	0.948
Missing	1 (0.6 %)	1 (0.7 %)	0 (0.0 %)	
Duration on ART (months)				
≤12 months	10 (5.73 %)	5 (3.3 %)	5 (20.8 %)	0.001
>12 months	165 (94.3 %)	146 (96.7 %)	19 (79.2 %)	
Median duration on ART (IQR) years	4.6 (2.4–6.4)	4.8 (2.7–6.4)	3.5 (1.5–6.6)	0.145
**Clinical characteristics**				
Admitted to the hospital in last 12 months				
No	166 (94.9 %)	144 (95.4 %)	22 (91.7 %)	
Yes	9 (5.1 %)	7 (4.6 %)	2 (8.3 %)	0.446
Admitted to the hospital for a chest infection/pneumonia in last 12 months				
No	171 (97.7 %)	149 (98.7 %)	22 (91.7 %)	
Yes	4 (2.3 %)	2 (1.3 %)	2 (8.3 %)	0.033

Laboratory analysis of the stool samples showed that 28/175 (16.0 %) were positive for possible ESBL-Es on initial screening. On confirmatory testing, 24 samples did contain an ESBL-E giving an estimated ESBL-E prevalence of 13.7 % (95 % CI 8.9–19.7) in this population. Of these, 23/24 (95.8 %) were identified as *E. coli* and 1/24 (4.2 %) as *K. pneumoniae*. The other four resistant isolates included three isolates identified as *E. coli* (not ESBL-producing) and one isolate identified as *Acinetobacter baumannii,* which was not further investigated. MALDI-TOF testing confirmed the identification of all organisms (data not shown). No CPE were detected.

Bivariate analysis to assess the factors associated with ESBL-E carriage found no demographic association with ESBL-E carriage. Independent association, however, was found between ESBL-E carriage and duration on ART (*P*=0.001) whereby participants on ART for less than 1 year had 7.7 times (95 % CI : 2.03–29.0) the odds of carrying an ESBL-E (Table 2). Similarly, participants admitted to hospital for a chest infection in the previous 12 months had 6.8 times the odds of carrying ESBL-Es (95 % CI : 0.9–50.6; *P*=0.033). These factors remained significant when considered in the final multivariate model when participants had eight times the odds of carrying ESBL-Es. Demographic and other clinical factors were included in the multivariate model despite *P*-values>0.2, and no significant association was observed even in the adjusted model.

**Table 2.** Univariate and multivariate analysis of factors associated with ESBL-E carriage

	Univariate OR (95 % CI)	*P*-value	Adjusted multivariate OR (95 % CI)	Final adjusted multivariate OR (95 % CI)
**Demographics**				
Gender				
Male	1		1	–
Female	1.33 (0.56–3.16)	0.516	1.31 (0.51–3.36)	
Age (years)				
6–11	1		1	
12–16	1.36 (0.57–3.27)	0.493	1.15 (0.44–2.96)	–
Median age (IQR) years		0.291		
Current CD4 count (cells µl^−1^)				
<350	1.81 (0.55–6.01)	0.330	1.08 (0.21–5.49)	–
≥350	1			
Median CD4 count (IQR)		0.548		
Current viral load (copies ml^−1^)				
≤50	1		1	
>50	1.03 (0.41–2.57)	0.948	0.93 (0.29–3.00)	–
Missing	–			
Duration on ART (years)				
≤1	7.68 (2.03–29.0)	0.003	8.19 (2.06–32.48)	8.47 (2.22–2.27)
>1	1		1	1
Median duration on ART (IQR) years		0.145		
**Clinical characteristics**				
Admitted to the hospital in last 12 months				
No	1			
Yes	1.87 (0.36–95.6)	0.453	–	–
Admitted to the hospital for a chest infection/pneumonia in last 12 months				
No	1			1
Yes	6.77 (0.907–50.587)	0.062	8.12 (0.98–67.17)	8.47 (1.12–64.07)

Results from AST on the ESBL-producers included 100 % resistance to co-trimoxazole, 91.6 % to ceftriaxone, 62.5 % to ciprofloxacin, 45.8 % to chloramphenicol and 20.8 % to gentamicin (see Table 3). All organisms were sensitive to amikacin and meropenem.

**Table 3.** Antibiotic susceptibility

ID	Species	MLST*	Resistance†	*β*-Lactamases	Antibiotic discs‡							
					TEM30	TZP36	MER	CN10	CIP5	W5	SXT25	C30
01	*E. coli*	48	AmpC§	CMY-2	S	S	S	S	S	R	R	S
02	*E. coli*	48	ESBL	CTX-M (gp|| 1), TEM-1	S	S	S	R	S	R	R	R
03	*E. coli*	131	AmpC§	DHA-1, TEM-1	S	S	S	R	R	R	R	S
04	*E. coli*	131	ESBL	CTX-M (gp 1), OXA-1	R	R	S	R	R	R	R	S
05	*E. coli*	131	ESBL	CTX-M (gp 1), OXA-1	S	I	S	S	R	R	R	S
06	*E. coli*	131	ESBL	CTX-M (gp 9)	S	S	S	S	R	R	R	S
07	*E. coli*	131	ESBL	CTX-M (gp 9)	S	S	S	S	R	R	R	S
08	*E. coli*	131	ESBL	CTX-M (gp 9), OXA-1, TEM-1	I	R	S	S	R	R	R	S
09	*E. coli*	131	ESBL	CTX-M (gp 9)	S	S	S	S	R	R	R	S
10	*E. coli*	131	ESBL	CTX-M (gp 9)	S	S	S	S	R	R	R	S
11	*E. coli*	131	ESBL	CTX-M (gp 9)	S	S	S	S	R	R	R	S
12	*E. coli*	393	ESBL	CTX-M (gp 1), TEM-1	S	S	S	R	R	R	R	S
13	*E. coli*	617	ESBL	CTX-M (gp 1), OXA-1	I	S	S	S	R	R	R	S
14	*E. coli*	656	ESBL	SHV-2a, TEM-1	S	S	S	S	S	R	R	S
15	*E. coli*	656	ESBL	SHV-2a, TEM-1; blaLEN-4	S	S	S	S	S	R	R	S
16	*E. coli*	1163	ESBL	CTX-M (gp 9), TEM-1	S	S	S	S	S	R	R	R
17	*E. coli*	1286	ESBL	CTX-M (gp 1)	S	S	S	S	S	R	R	R
18	*E. coli*	1589	ESBL	CTX-M (gp 1)	S	S	S	S	R	R	R	R
19	*E. coli*	1589	ESBL	CTX-M (gp 1)	S	S	S	S	R	R	R	R
20	*E. coli*	2461	ESBL	CTX-M (gp 9)	S	S	S	S	S	R	R	R
21	*E. coli*	3036	AmpC§	DHA-1	S	S	S	S	S	R	R	S
22	*E. coli*	ST167 (SLV)	ESBL	CTX-M (gp 1), OXA-1, TEM-1	R	R	S	R	R	R	R	R
23	*E. coli*	ST202 (SLV)	ESBL	CTX-M (gp 9)	S	S	S	S	S	R	R	R
24	*E. coli*	ST2601 (SLV)	ESBL	CTX-M (gp 1)	S	S	S	S	S	R	R	R
25	*E. coli*	ST2753 (SLV)	ESBL	CTX-M (gp 9), TEM-1	S	S	S	S	S	R	R	R
26	*E. coli*	ST224	ESBL	CTX-M (gp 1)	S	S	S	S	R	R	R	R
27	*K. pneumoniae*	340	ESBL	CTX-M (gp 1), OXA-1; SHV-11;TEM-1; blaLEN-11	R	R	S	R	R	R	R	S

*Multi-locus sequence type.

†Phenotypic mechanism of resistance.

‡Tem30, temocillin 30 µg; TZP36, piperacillin-tazobactam 36 µg; Mer, meropenem 10 µg; CN10, gentamicin 10 µg; CIP5, ciprofloxacin 5 µg; W5, trimethoprim 5 µg; SXT25, co-trimoxazole 25 µg; C30, chloramphenicol 30 µg.

§Plasmid-associated AmpC.

||Group.

The 27 isolates (i.e. 23 ESBL-positive *E. coli*, one ESBL-positive *K. pneumoniae* and three *E. coli* with AmpC enzymes) were genetically diverse, represented by 16 different MLSTs (15 STs in *E. coli*, 1 *K. pneumoniae*). Molecular characteristics of the isolates relevant to this paper are displayed in Table 3. The detail of these CTX-M genes is part of an analysis of flows of resistance genes and bacterial groups, which is beyond the scope of this work. The flow of CTX-M genes, bacterial serogroups, clades and sub-clades will be described in a subsequent phylogeography-focused analysis. The *E. coli* were predominated by nine ST131 isolates with the remainder comprised of two isolates each of STs 48, 656 and 1589, and 11 singletons. The ESBLs were predominated by CTX-M enzymes, comprising mainly group-9 CTX-Ms (six CTX-M-27 and four CTX-M-14) followed by seven CTX-M-15 and four CTX-M-3, both in group 1 (Table 3). Three isolates had acquired AmpC-type β-lactamases (two DHA-1 and one CMY-2). Group 1 and group 9 CTX-M ESBLs were disseminated across multiple STs and, in the case of group 1, found even in a different species (*K. pneumoniae*).

A duration of ART treatment of less than 1 year was associated with a significantly higher ESBL-E carriage prevalence (20.8 % versus 3.3 %, *P*=0.001), as was having been admitted to hospital for a chest infection in the past 12 months (8.3 % versus 1.3 %, *P*=0.033; Table 1). There was no association between ESBL-E carriage and sex, age, CD4 count or viral load.

## Discussion

The key finding of this study was an ESBL-E carriage prevalence of 13.7 % among children attending routine outpatient HIV care. This is lower than the recently (2016) reported outpatient setting carriage prevalence estimate of 59 % (79/134) in rectal swabs from healthy young children in the Central African Republic and higher than the 7.4 % in nasopharygeal swabs from HIV-infected children in Cape Town. Among the ESBL-E isolates, there was a high level of resistance to co-trimoxazole, ciprofloxacin, chloramphenicol and gentamicin. Participants enrolled in the current study were often repeatedly exposed to inpatient and outpatient environments and to have received multiple courses of antibiotics throughout their short lives due to their chronic immunodeficient state. Carriage was associated with having started ART recently and having been hospitalized with a chest infection in the previous 12 months. Unfortunately, reliable information on previous antibiotic usage was not available and parents were often unsure of the exact treatment their child had received.

Two recent reviews on MDRO prevalence in Africa reported ESBL-E prevalence rates ranging from 1.3–22.8 % in urine and 0.7–75.8 % in blood [5, 17]. Unfortunately the analysis was not stratified by region. Another study from Malawi reported a very low prevalence of 0.7 % ESBL-Es in bloodstream infections in 2005 in adult and paediatric medical wards in a large government tertiary hospital in Blantyre [18]. This was at a time when ceftriaxone, the only third generation cephalosporin available in many African countries, was not yet widely available in Malawi. ESBL-E prevalence is likely to have increased throughout Africa in line with the reported global ESBL-E increase worldwide [2, 5, 7].

Being admitted to hospital within the previous 12 months for a chest infection was associated with ESBL-E carriage (*P*=0.033). This points to the possibility of having acquired the ESBL-E while in hospital. The other potential significant risk factor was having been on ART for less than 1 year (*P*=0.002). These children are likely to have been immunosuppressed more recently and to have received antibiotics and/or to have recent contact with healthcare services, compared to children well established on ART for years.

The ESBL-Es identified in this study were resistant to multiple classes of antibiotics. All isolates were resistant to co-trimoxazole with less resistance to ciprofloxacin (60 %), chloramphenicol (45 %), gentamicin (20 %) and temocillin (12 %), and none to amikacin. The level of resistance reflects the usage of these antibiotics in the public sector in Zimbabwe. All HIV-infected individuals receive long-term co-trimoxazole for prophylaxis, and WHO guidelines recommend continuation of co-trimoxazole in children even following immune reconstitution with ART [19]. Ciprofloxacin is frequently used in the community setting and is the antibiotic of choice in children presenting with diarrhoeal illnesses and fever in primary care [20]. Ceftriaxone and chloramphenicol are used interchangeably for meningitis treatment depending on availability in the public sector. While ceftriaxone is the first-line antibiotic for most infections requiring antibiotic treatment in a hospital setting, meropenem is not available in the public sector in Zimbabwe, which may account for the lack of carbapenemase producers.

Our observation of multiple cases affected by ST131 *E. coli* aligns with the predominance of this ST in many other parts of the world [21] and its enhanced virulence, and constitutes the first report, to our knowledge of this lineage in Zimbabwe. Moreover, all of the ESBL *E. coli* we observed, including the ST131 isolates, encoded ESBLs alongside multiple other resistances (co-trimoxazole, aminoglycosides and fluoroquinolones). Concurrent antimicrobial resistance limits treatment options and is known to be typically encoded on plasmids and transposons that are capable of intra- and inter-species spread. If carriage in these children were to result in urinary or bloodstream infections, gentamicin would be the only available antibiotic with a reasonable chance of treatment success.

The study was conducted in the public sector among asymptomatic and previously unstudied HIV-infected children and adolescents. Multiple standardized and well-established methods were used to identify ESBL-E phenotypically and genotypically. However, the sample size was small resulting in reduced power to identify risk factors. A limitation of this study was that no comparison was made with HIV-negative children to ascertain whether ESBL-E carriage was associated with HIV status. Thus whether the prevalence of 13.7 % is specific to this patient population or due to a high carriage rate in the community is unknown and requires further investigation. Variables such as self-reported hospitalization are subject to recall bias, although we attempted to confirm contact with health services from patient-held clinical records. Participants may have been hospitalized for other infections, but as this study was embedded in a study investigating chronic lung disease, only hospitalisations for lung-related infections were recorded. However, respiratory tract infection is the most common infection among HIV-infected children, including among those who are immunocompetent.

The ESBL-E prevalence identified in this study carries several important implications. First, the carriers themselves are at risk of becoming infected. Gram-negative sepsis due to ESBL-Es remains largely untreated in this setting and is associated with very high mortality. Second, these children are in recurrent contact with health services and, as such, the ESBL-E carriage has implications for infection control. Finally, this study is a reminder of how the use of ceftriaxone, a broad-spectrum third-generation cephalosporin widely used as a first-line antibiotic for sepsis and meningitis throughout Sub-Saharan Africa [22], might favour the spread of ESBL-Es. Limited diagnostic capacity for identifying the source of sepsis and the causative organism and its susceptibilities contribute to the widespread use of broad-spectrum cephalosporins in this region. In order to assess the prevalence of MDROs in Sub-Saharan Africa, it is important to address the severe limitations in microbiological diagnostic facilities for their detection and to enable targeted antimicrobial therapy and minimize the overuse of broad-spectrum empiric antibiotics.
